# Genome Sequence of Pseudomonas azelaica Strain Aramco J

**DOI:** 10.1128/genomeA.00037-15

**Published:** 2015-03-05

**Authors:** Magdy El-Said Mohamed, José L. García, Igor Martínez, Carlos del Cerro, Juan Nogales, Eduardo Díaz

**Affiliations:** a R&DC, Saudi Aramco, Dhahran, Saudi Arabia; b Environmental Biology, Centro de Investigaciones Biológicas, Consejo Superior de Investigaciones Científicas, Madrid, Spain

## Abstract

We report here the draft genome sequence of Pseudomonas azelaica strain Aramco J (7.3 Mbp; GC content, 61.9%), one of the few bacteria that can completely mineralize different hydroxybiphenyls, e.g., 2-hydroxybiphenyl, 2,2′-dihydroxybiphenyl, and 3-hydroxybiphenyl. The findings obtained from its genome annotation suggest that this strain becomes a useful biocatalyst for aromatic bioconversions.

## GENOME ANNOUNCEMENT

Hydroxybiphenyls, such as 2-hydroxybiphenyl (2-HBP), are of great importance in the metabolism of toxic biphenyls, such as polychlorinated biphenyls, and 2-HBP is also a main byproduct of oil biodesulfurization produced through microbial conversion of dibenzothiophene (1). Moreover, 2-HBP is a bulk chemical with biocidal properties that is primarily used as an agricultural fungicide and therefore can be found in persistent low quantities in sewage effluents (2). However, bacterial degradation of 2-HBP is an uncommon trait, and only a few bacteria are known to completely metabolize 2-HBP (3, 4). Here, we report the genome sequence of a bacterium, the Pseudomonas azelaica Aramco J strain, isolated from an oil-contaminated soil sample from Abu Ali Island, Saudi Arabia, that uses 2-HBP, as well as other hydroxybiphenyls such as 2,2′-dihydroxybiphenyl and 3-hydroxybiphenyl, as the only carbon source.

The genome of the Aramco J strain has been sequenced using the 316-chip and 400-bp chemistry Ion Torrent PGM platform per the manufacturer’s instructions. Preliminary assembly of raw lectures (528 Mbp sequence) was performed using Newler software from Roche. This assembly was manually revised and improved, obtaining a quality draft of 91 contigs. The genome was structurally and functionally annotated using RAST (5), an automated genome annotation system, and the functions, names, and general properties of the gene products were predicted using this method. The Aramco J genome (7.3 Mbp) is highly similar (99% nucleotide identity) to that of P. azelaica HBP1 (6), indicating that this strain is a new member of the P. azelaica species. However, P. azelaica strain Aramco J lacks a 75-kb DNA fragment that is present in strain HBP1 in a megaplasmid. The smaller megaplasmid of strain Aramco J shows significant similarity to the pOZ176 IncP-2 megaplasmid of Pseudomonas aeruginosa (7), and its average GC content (57%) is lower than that of the whole genome (61.9%).

The *hbp* genes involved in the conversion of 2-HBP (and 2,2′-dihydroxybiphenyl) to benzoate (salicylate) and 2-hydroxy-pentadienoate (4) are located within an integrative-conjugative element (ICEhbp) similar to that found in P. azelaica HBP1 (6). Next to the *hbp* genes are located the *sal* and *dmp* genes encoding a salicylate monooxygenase and a catechol *meta*-cleavage pathway for the metabolism of salicylate and 2-hydroxy-pentadienoate, respectively. Other catabolic functions present in ICEhbp are the *pob-pca* genes for 4-hydroxybenzoate degradation via the β-ketoadipate *ortho*-cleavage pathway, as well as genes likely involved in channeling aromatic alcohols with methoxy substituents, e.g., coniferyl alcohol, to the β-ketoadipate pathway. At least two different *ben-cat* clusters for benzoate degradation, and the *paa* and *hmg* clusters for phenylacetate and homogentisate degradation, respectively, can be identified outside the ICEhbp element.

Given the catabolic potential of the P. azelaica strain Aramco J toward aromatic compounds, as revealed by the genome information reported here, and its unique tolerance to high 2-HBP concentrations (up to 15 g/liter), this strain becomes an interesting host for engineering recombinant biocatalysts to be used in biotechnological processes such as biodesulfurization (1).

### Nucleotide sequence accession numbers.

This whole-genome shotgun project has been deposited at DDBJ/EMBL/GenBank under the accession number JUEH00000000. The version described in this paper is version JUEH01000000.
